# Limitations of a Commercial Assay as Diagnostic Test of Autoimmune Encephalitis

**DOI:** 10.3389/fimmu.2021.691536

**Published:** 2021-06-29

**Authors:** Raquel Ruiz-García, Guillermo Muñoz-Sánchez, Laura Naranjo, Mar Guasp, Lidia Sabater, Albert Saiz, Josep Dalmau, Francesc Graus, Eugenia Martinez-Hernandez

**Affiliations:** ^1^ Immunology Department, Centre Diagnòstic Biomèdic, Hospital Clínic, Barcelona, ​​Spain; ^2^ Neuroimmunology Program, Institut d’Investigacions Biomèdiques August Pi i Sunyer (IDIBAPS), Barcelona, ​​Spain; ^3^ Neurology Department, Hospital Clinic, and University of Barcelona, Barcelona, ​​Spain; ^4^ Centro de Investigación Biomédica en Red, Enfermedades Raras (CIBERER), Madrid, Spain; ^5^ Neurology Department, University of Pennsylvania, Philadelphia, PA, United States; ^6^ Catalan Institution of Research and Advanced Studies (ICREA), Barcelona, Spain

**Keywords:** neuronal antibodies, brain immunohistochemistry, diagnostic test, autoimmune encephalitis (AE), immunofluorescent assay

## Abstract

Detection of neuronal surface antibodies (NSAb) is important for the diagnosis of autoimmune encephalitis (AE). Although most clinical laboratories use a commercial diagnostic kit (Euroimmun, Lübeck, Germany) based on indirect immunofluorescence on transfected cells (IIFA), clinical experience suggests diagnostic limitations. Here, we assessed the performance of the commercial IIFA in serum and CSF samples of patients with suspected AE previously examined by rat brain immunohistochemistry (Cohort A). Of 6213 samples, 404 (6.5%) showed brain immunostaining suggestive of NSAb: 163 (40%) were positive by commercial IIFA and 241 (60%) were negative. When these 241 samples were re-assessed with in-house IIFA, 42 (18%) were positive: 21 (9%) had NSAb against antigens not included in the commercial IIFA and the other 21 (9%) had NSAb against antigens included in the commercial kit (false negative results). False negative results occurred more frequently with CSF (29% vs 10% in serum) and predominantly affected GABA_B_R (39%), LGI1 (17%) and AMPAR (11%) antibodies. Results were reproduced in a separate cohort (B) of 54 AE patients with LGI1, GABA_B_R or AMPAR antibodies in CSF which were missed in 30% by commercial IIFA. Patients with discordant GABA_B_R antibody results (positive in-house but negative commercial IIFA) were less likely to develop full-blown clinical syndrome; no significant clinical differences were noted for the other antibodies. Overall, NSAb testing by commercial IIFA led to false negative results in a substantial number of patients, mainly those affected by anti-LG1, GABA_B_R or AMPAR encephalitis. If these disorders are suspected and commercial IIFA is negative, more comprehensive antibody studies are recommended.

## Introduction

Detection of antibodies to neuronal surface proteins and synaptic receptors is important to establish a definitive diagnosis of autoimmune encephalitis (1). Well-characterized clinical syndromes associate with specific antibodies, and previously unrecognized neurological diseases are currently defined by the corresponding neuronal surface antibody, such as anti-NMDAR encephalitis or anti-LGI1 encephalitis, which are the most frequent antibody-mediated encephalitis (2). Commercial diagnostic kits using transfected cells that express the most common neuronal surface antigens are widely available, and allow rapid antibody testing in clinical laboratories (3). Most clinical laboratories worldwide use the same commercial indirect immunofluorescence assay (IIFA) whereas rat brain immunohistochemistry is only performed in a few specialized centers (4). However, there are no studies comparing the performance of commercial IIFA with the combination of rodent brain immunohistochemistry and IIFA as used in the initial description of most neuronal surface antibodies. There is preliminary data suggesting that the sensitivity of commercial IIFA, particularly for LGI antibodies in cerebrospinal fluid (CSF), may be low, and false positive results may occur particularly when serum is used at high concentration (4, 5). Moreover, commercial diagnostic kits contain a limited number of antigens (up to 6 specificities) and some less frequent or recently described antigens are not included. For these reasons, the use of commercial IIFA as the only method to diagnose autoimmune encephalitis probably misses the detection of otherwise well-characterized and relevant antibodies. This can have important implications such as overlooking the presence of tumors typically associated with some of the non-detected antibodies, or not giving immunotherapy to patients with unrecognized autoimmune encephalitis. Here, we assessed the diagnostic value and limitations of a commercial kit for the detection of neuronal surface antibodies in the serum and CSF of patients with autoimmune encephalitis.

## Patients and Methods

### Patients and Samples

We prospectively examined 6213 serum and CSF samples from patients referred to our diagnostic lab for detection of antibodies against neuronal surface antigens from October-2016 to October-2020 (Cohort A). Samples were screened with rat brain immunohistochemistry and results were examined by two independent observers. Samples showing positive immunostaining suggestive of a neuropil antibody were first studied with commercial IIFA (6). Samples that were positive on brain immunohistochemistry (with a pattern of staining suggesting a neuronal surface antibody) but negative on commercial IIFA were later studied with in-house IIFA. Clinical data was reviewed in all cases with available information. To further assess the performance of the commercial IIFA, we retrospectively studied 54 consecutive CSF samples from patients with encephalitis and LGI1 (n=12), AMPAR (n=19) or GABA_B_R (n=23) antibodies confirmed by brain immunohistochemistry and in-house IIFA (Cohort B).

### Rat Brain Immunohistochemistry

Tissue immunohistochemistry was performed as previously described (7). Briefly, adult Wistar rats were euthanized in a CO_2_ chamber and the brain was removed without previous tissue perfusion. Brains were sagittally split in two hemispheres, immersed in 4% paraformaldehyde for 1h at 4°C, cryoprotected with 40% sucrose for 48h, and snap frozen in chilled isopentane. Frozen sections were air-dried for 30 min and sequentially treated with hydrogen peroxide 30% in PBS for 15 minutes. Brain sections were blocked with 5% normal goat serum in PBS for 1h at room temperature and incubated with patients’ sera (diluted 1:200) or CSF (1:2) overnight at 4°C. Biotinylated goat anti-human IgG (Vector Labs, Burlingame, CA, 114 USA) was added for 2 h, followed by incubation with the avidin–biotin immunoperoxidase complex (Vector Labs, Burlingame, CA, 114 USA) for 1 h. The reaction was developed with 0.05% diaminobenzidine (Sigma, St. Louis, MO, USA).

### Indirect Immunofluorescence Assays

Samples that produced a neuropil immunostaining on rat brain immunohistochemistry were subsequently examined with two types of IIFAs: 1) the Autoimmune Encephalitis Mosaic 6 kit (Euroimmun, Lübeck Germany), following manufacturer’s instructions and recommended dilutions (undiluted CSF and 1:10 serum), to test IgG antibodies against N-methyl-D-aspartate (NMDA) receptor (GluN1), α-amino-3-hydroxyl-5-methyl-4-isoxazole-propionate (AMPA) receptor (GluA1, GluA2), gamma-aminobutyric (GABA) B receptor (B1 and B2 subunits), contactin-associated protein-like 2 (CASPR2), leucine-rich glioma-inactivated protein 1 (LGI1) and dipeptidyl-peptidase 6 (DPPX), 2) in-house IIFAs in which HEK293 cells were transfected with DNA constructs to express the following antigens: NMDA receptor (GluN1, GluN2), AMPA receptor (GluA1, GluA2), GABA_B_ receptor (B1, B2), CASPR2, LGI1 (with and without disintegrin and metalloproteinase domain-containing protein 23 [ADAM23] co-transfection), GABA_A_ receptor (α1, β3), metabotropic glutamate receptors mGluR1, mGluR2, mGluR5, Ig-Like Domain-Containing Protein 5 (IgLON5) or Seizure 6-like protein 2 (SEZ6L2) as previously described (7–17). Briefly, sera and CSF were diluted in PBS-1% BSA (1:2 CSF and 1:40 serum) and incubated with stored pre-fixed transfected cells overnight, and with an anti-human IgG antibody conjugated with AF488 or AF594 (Invitrogen) for 1 hour. Live, non-fixed transfected cells were used for detection of GPI-LGI1 (without ADAM23), GABA_A_ receptor, mGluR1-2-5, IgLON5 and SEZ6L2 antibodies. IIFA results were observed in an Axio-Imager 2 microscope (Zeiss, Germany).

The study was approved by the ethics committee of Hospital Clínic of Barcelona. Patients’ samples were coded and clinical information was anonymized prior to analysis. Written inform consent was not required as the study was observational, and the detection of neuronal surface antibodies was requested as part of the routine diagnostic work-up.

## Results

**Figure 1:**
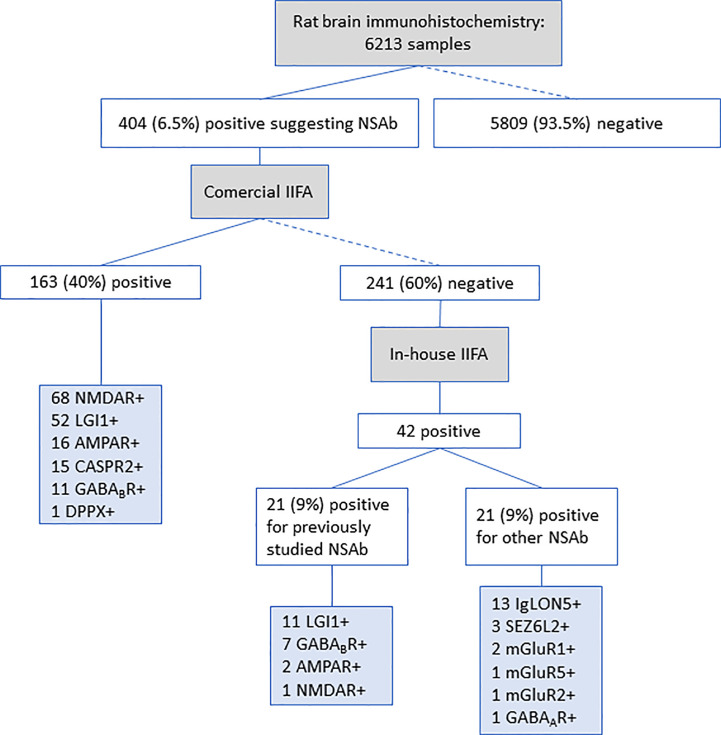
Antibody detection in patients from Cohort A. Workflow used to identify IgG neuronal surface antibodies in a cohort of 6231 samples. IIFA, Indirect immunofluorescent assay; NSAb, Neuronal surface antibodies.

**Figure 2:**
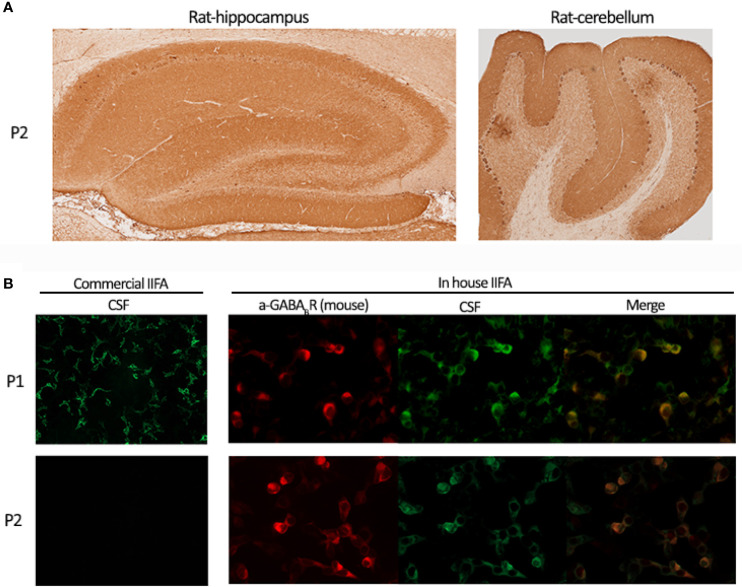
Discrepancies identifying GABA_B_R antibodies by IIFA. **(A)** One of the two patients’ CSF (P2) demonstrating GABA_B_R immunoreactivity on rat brain immunohistochemistry. Hippocampus (left panel) and cerebellum (right panel) staining patterns. **(B)** Upper panels show patient 1’s CSF (P1) reactivity on commercial IIFA and in-house IIFA GABA_B_R transfected cells; lower panels show patient 2’s CSF (P2) reactivity on commercial IIFA (negative staining) and in-house IIFA (positive staining) GABAbR transfected cells.

In Cohort A, 404 (6.5%) of the 6213 samples (222 sera, 182 CSF) showed a positive staining on brain immunohistochemistry suggesting the presence of neuronal surface antibodies. These 404 samples were analyzed by the commercial IIFA and 163 (40%) resulted positive for IgG against one of the included antigens: 68 (42%) for NMDAR [27 sera/41 CSF], 52 (32%) for LGI1 [26 sera/26 CSF], 16 (10%) for AMPAR [11 sera/5 CSF], 15 (9%) for CASPR2 [9 sera/6 CSF], 11 (7%) for GABA_B_R [6 sera/5 CSF] and 1 (1%) for DPPX [serum] antibodies (**Figure 1**). Samples with positive brain immunohistochemistry and negative commercial IIFA results (241 samples) were further analyzed for neuronal surface antibodies by in-house IIFA. We performed the most suitable antigen specific assay depending on the immunostaining pattern and/or the clinical phenotype. Twenty-one (9%) of these 241 samples were positive for antibodies against antigens not included in the commercial kit (13 IgLON5, 3 SEZ6L2, 2 mGluR1, 1 mGluR2, 1 mGluR5, and 1 GABA_A_R) (**Figure 1**). Additionally, 21 (9%) of the 241 commercial IIFA-negative samples showed a positive result on the in-house IIFA for antigens included in the commercial kit: 11 LGI1 (4 sera/7 CSF), 7 GABA_B_R (1 serum/6 CSF), 2 AMPAR (2 CSF), and 1 NMDAR (serum) (**Figures 1** and **2**). These 21 samples were considered as false negative results of the commercial IIFA. The frequency of false-negatives was 39% (7/18) for GABA_B_R, 17% (11/63) for LGI1, 11% (2/18) for AMPAR, and 1.4% (1/69) for NMDAR antibodies. Among 41 patients with paired serum and CSF samples we obtained false negative results in 7 (17%): antibodies were not detected in one sample by the commercial IIFA in 5/15 patients with LGI (3 in serum, 2 in CSF) and 1/5 with AMPAR antibodies (in CSF), and not detected in serum and CSF in 1/3 patients with GABA_B_R antibodies (**Supplementary Table**). The commercial kit failed to detect GABA_B_R, LGI1 and AMPAR antibodies more frequently in CSF than serum (29% [15/51] of CSF samples compared to 10% [5/48], respectively, p=0.024).

Considering that CSF samples gave more discordant results between commercial and in-house IIFAs and CSF antibody detection is crucial for the diagnosis of autoimmune encephalitis, we assessed 54 additional CSF samples of patients with encephalitis and GABA_B_R, LGI1 or AMPAR antibodies, confirmed by brain immunohistochemistry and in-house IIFA, and retested them by commercial IIFA (Cohort B). The commercial kit failed to detect antibodies in 16 (30%) samples: 4/12 (33%) with LGI1, 7/23 (30%) with GABA_B_R, and 5/19 (26%) with AMPAR antibodies. Overall, we obtained a similar frequency of CSF false negative results in both cohorts: 5/51 (29%) from cohort A and 16/54 (30%) from cohort B.

**Figure 3:**
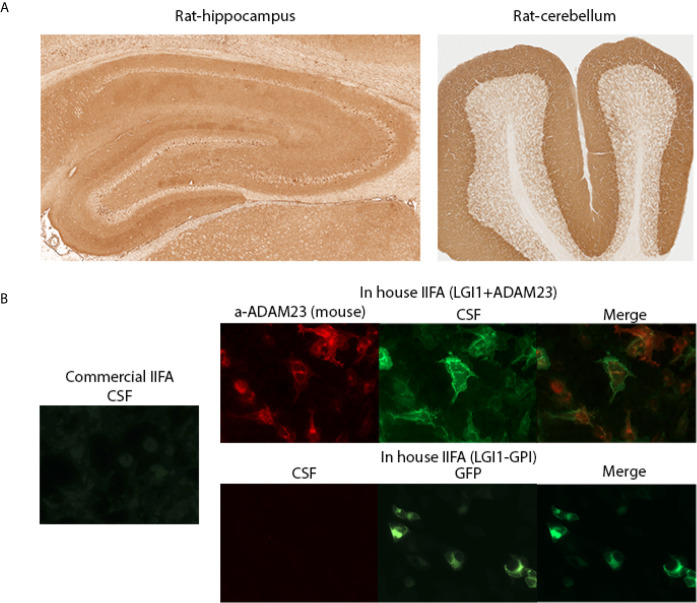
Discrepancies identifying LGI1 antibodies by IIFA. **(A)** A patient’s CSF demonstrating LGI1 immunoreactivity on rat brain immunohistochemistry. Hippocampus (left panel) and cerebellum (right panel) staining patterns. **(B)** Left panel shows patient’s CSF reactivity on commercial IIFA LGI1 transfected cells (negative staining); Right panels show CSF reactivity on in-house IIFAs using LGI1 plus ADAM23 transfected cells (upper panels; positive staining) and GPI-LGI1 transfected cells (lower panels, negative staining). GPI: glycosyl-phosphatidylinositol anchored LGI1 (to display the protein on the cell surface).

As LGI1 cell transfection differed between the commercial kit and our in-house IIFA (co-transfected with ADAM23), we used a modified in-house GPI-LGI1 IIFA (surface-expressing full-length LGI1 construct, without ADAM23, as reported) (18). We tested again the 11 discordant commercial/in-house IIFA CSF samples of Cohort A (7), and Cohort B (4), and all were found negative by GPI-LGI1 IIFA, suggesting that LGI1 antibody detection in the CSF requires ADAM23 co-expression (**Figure 3**). Samples were also negative on cells transfected only with ADAM23 (data not shown). We found no alternative explanation for the lower detection of GABA_B_R and AMPAR antibodies with the commercial IIFA.

Next, we reviewed the demographic and baseline clinical features of patients with GABA_B_R/LGI1/AMPAR antibodies in the CSF of Cohort A (51 samples of 48 patients; 40 with available information) and Cohort B (54 patients). **Table 1** shows the comparison between patients with concordant (66) and discordant (28) commercial/in-house IIFA results for each antibody. We found that patients with discordant GABA_B_R antibody detection (positive by in-house and negative by commercial IIFA) developed less frequently the full-blown clinical encephalitis syndrome than those with a concordant detection (positive by both IIFAs). In the discordant group 3 patients had refractory epileptic seizures and 1 had chorea (4/11) when their samples were sent for study, whereas in the concordant group 1/21 had refractory seizures (p=0.011). Additionally, brain MRI from patients with discordant GABA_B_R and AMPAR IIFA results were more often normal at the time antibodies were determined (**Table 1**). Other clinical features (age, gender or tumor association) were not different between patients with concordant and discordant antibody results.

**Table 1:** Clinical features of patients with antibodies in the cerebrospinal fluid, and comparison between those with concordant and discordant results.

	Concordant in-house/commercial IIFA	Discordant in-house/commercial IIFA	p value
**LGI1+**	27 (19 [A] + 8 [B])	10 (6 [A] + 4 [B])	
Median age	65 y (range: 43-88)	59 y (range: 36-83)	0.352
Male sex	15 (55%)	5 (50%)	1.00
Encephalitis	23 (85%) *3 seizures, 1 Morvan’s syndrome	10 (100%)	0.557
Abnormal MRI	11/16 (69%)	4/6 (67%)	1.00
Tumor	2 (7%) **1 breast cancer, 1 thymoma	1 (10%) **1 colon adenocarcinoma	1.00
**GABA_B_R+**	21 (5 [A] + 16 [B])	11 (4 [A] + 7 [B])	
Median age	63.5 y (range: 24-78)	57 y (range: 36-81)	0.942
Male sex	12 (57%)	5 (45%)	0.712
Encephalitis	20 (95%) *1 refractory seizures	6 (55%) *4 refractory seizures, 1 chorea	**0.011**
Abnormal MRI	11/16 (69%)	2/9 (22%)	**0.041**
Tumor	9 (43%) **5 lung cancer, 2 neuroendocrine of unknown origin, 1 gastric, 1 vesical	4 (36%) ** 3 lung, 1 breast cancer	1.00
**AMPAR+**	18 (4 [A] + 14 [B])	7 (2[A] + 5[B])	
Median age	58 y (range: 21-83)	54 y (range: 27-81)	0.981
Male sex	9 (50%)	3 (43%)	1.00
Encephalitis	17 (94%) *1 refractory seizures	6 (86%) *1 refractory seizures	0.490
Abnormal MRI	10/12 (83%)	1/5 (20%)	**0.028**
Tumor	11 (61%) **5 lung cancer, 4 thymoma, 1 breast, 1 teratoma	4 (57%) **3 lung cancer, 1 teratoma	1.00

A, patients from Cohort A; B, patients from Cohort B; IIFA, indirect immunofluorescence assay; MRI, magnetic resonance image; y, years.

*Other clinical presentations; **Tumor types. In bold: Statistical significance p < 0.05.

## Discussion

Our study shows that neuronal surface antibody detection using only the commercial IIFA has limitations in the diagnosis of autoimmune encephalitis. Among 241 samples that were positive by brain immunohistochemistry but negative by the commercial kit, we found 42 (18%) that had well-defined neuronal surface antibodies using our in-house IIFA. Half of these samples had antibodies against antigens not included in the commercial kit. However, the remaining 50% of samples were considered false negative results, as the in-house IIFA detected antibodies that should had been detected by the commercial IIFA. False negative results were more common in CSF (29% for CSF vs. 10% for serum), and particularly occurred for the antibodies against GABA_B_R (39% of cases), LGI1 (17%) and AMPAR (11%). Similar results were obtained in a separate series of 54 CSF samples (Cohort B) from patients with LGI1, GABA_B_R or AMPAR antibodies (detected by brain immunohistochemistry and in-house IIFA) showing that the commercial kit failed to detect 33% of LGI1, 30% of GABA_B_R, and 26% of AMPAR antibodies.

These results agree with previous reports based on commercial IIFA, describing a higher sensitivity for LGI1 and GABA_B_R antibody detection in the serum of patients with autoimmune encephalitis. For example, a study assessing 256 patients with LGI1/CASPR2 antibodies with the same commercial kit found lower sensitivity in CSF than in serum testing. Among 196 patients with LGI antibodies the authors found commercial IIFA positivity in 63% of CSF samples (24 of 38), and recommended that serum should be tested for determination of LGI1 antibodies by IIFA (19). In a second series of 38 patients with anti-LGI1 encephalitis, CSF was available for testing in 17 patients and only 9 (53%) were positive whereas all sera tested positive on the commercial IIFA (20).

A probable reason for false negative results in LGI1 antibody commercial testing is that the commercial kit does not use ADAM23 co-transfection (a presynaptic protein that forms a complex with LGI1 and interacts with voltage-gated potassium channels Kv1.1). When we re-tested the 11 CSF samples with discordant commercial/in-house LGI1 antibody results using GPI-LGI1 IIFA (cells transfected only with LGI1) all of them were found negative. An explanation could be that the recognition of some LGI1 target epitopes by CSF antibodies is improved when ADAM23 (a protein that normally interacts with LGI1) is co-expressed. The reasons for the disparity in the results of GABA_B_R and AMPAR antibody testing between commercial and in-house IIFAs are unclear. In a study reporting 32 patients with GABA_B_R encephalitis, antibody detection by commercial IIFA was less sensitive in CSF (16/20 positive) than in serum (29/30 positive). CSF results were slightly improved using in-house IIFA (18/20 positive) and with a modified assay co-transfecting the intracellular accessory protein of the B2 subunit of the GABA receptor potassium channel tetramerization domain-containing 16 (KCTD16) (20/20 positive) (21). Additionally, KCTD16 antibodies were identified in 72% of patients with anti-GABA_B_R encephalitis, using in-house IIFA on cells transfected only with KCTD16, and their presence indicated a higher association with lung cancer.

Finally, we compared the general clinical features between patients with concordant and discordant CSF commercial/in-house IIFA results focusing on the three antibodies that were more frequently misdiagnosed. Overall, patients with LGI, GABA_B_R or AMPAR antibodies had clinical features of the encephalitis typically associated with the corresponding antibody (median age 56-62 years, slight male predominance in anti-LGI1 and anti-GABA_B_R, and higher tumor association in anti-GABA_B_R and anti-AMPAR, 40-60%) regardless of the negative findings with the commercial kit (7, 10, 11). Patients with false GABA_B_R antibody results on the commercial kit had a higher frequency of refractory seizures as main clinical presentation, without prior cognitive or behavioral changes, but were not different in demographic characteristics or frequency of paraneoplastic cases.

A limitation of our study is that we did not evaluate the specificity of the commercial IIFA. Rat brain immunohistochemistry was used as a first step for neuronal surface antibody screening and all samples tested by commercial or in-house IIFAs had positive reactivity on tissue. In the case of NMDAR antibodies, detection by immunohistochemistry is known to be more sensitive than IIFA and previous studies found that the combination of rat brain immunohistochemistry and IIFA improved diagnostic accuracy in the evaluation of neuronal surface antibodies (5, 22). Several studies using commercial kits have reported NMDAR antibodies in serum of patients with many diseases different from anti-NMDAR encephalitis as well as in healthy persons (23–25). We did not systematically test all samples received by commercial IIFA so we do not know the frequency of false positive NMDAR antibody results that occur when the commercial kit findings are not confirmed by other techniques (rat brain immunohistochemistry or in house IIFA). Another limitation is the low frequency of some antibodies, such as DPPX, for which the diagnostic value of the commercial kit could not be assessed.

The main message of our study is that the commercial IIFA for autoimmune encephalitis leads to false negative results in a substantial number of patients, especially when CSF is used, and predominantly for LGI1, GABA_B_R and AMPAR antibodies. Overall, the implications are important given that 1) anti-LGI1 encephalitis is the most common encephalitis in adults, but up to 13% develop cognitive impairment without criteria of encephalitis (26). A false negative result in these patients may lead to erroneously rule out the diagnosis; 2) for antibodies such as GABA_B_R and AMPAR the lack of detection may represent missing an underlying tumor (lung cancer, breast cancer or thymoma); 3) delaying or not giving appropriate immunotherapy may impact patients’ outcome; and 4) studies on incidence, prevalence and biology of encephalitis, and recommendations about diagnosis are frequently based on results using commercial kits, and not on the real data of the disease when antibodies are comprehensively tested and results validated. Future studies investigating autoimmune encephalitis should consider these limitations. In case of negative results with the commercial kit, we recommend to extend the study using brain immunohistochemistry and in-house IIFA.

## Data Availability Statement

The raw data supporting the conclusions of this article will be made available by the authors, without undue reservation.

## Ethics Statement

The study was approved by the ethics committee of Hospital Clínic of Barcelona. Patients’ samples were coded and clinical information was anonymized prior to analysis. Written inform consent was not required as the study was observational, and the detection of neuronal surface antibodies was requested as part of the routine diagnostic work-up. Written informed consent for participation was not required for this study in accordance with the national legislation and the institutional requirements.

## Author Contributions

EM-H designed the study and wrote the manuscript in collaboration with RR-G, AS, JD, and FG. RR-G, GM-S, LN, LS, MG, and EM-H performed laboratory work and/or data analysis. All authors contributed to the article and approved the submitted version.

## Funding

Fondo Europeo de Desarrollo Regional (FEDER) – Instituto de Salud Carlos III (Juan Rodés grant JR17/00012, EM-H; FIS PI20/00197, JD and Proyecto Integrado de Excelencia 16/00014, JD); Resident award “Josep Font” by Hospital Clínic Research, Innovation and Education Departments (MG); La Caixa Foundation (JD); Edmond J Safra Foundation (JD); Fundació CELLEX (JD) and Red Española de Esclerosis Múltiple RD16/0015/0002, RD16/0015/0003 (AS).

## Conflict of Interest

JD receives royalties from Athena Diagnostics for the use of Ma2 as an autoantibody test and from Euroimmun for the use of NMDA, GABAB receptor, GABAA receptor, DPPX and IgLON5 as autoantibody tests. FG holds a patent for the use of IgLON5 as an autoantibody test.

The remaining authors declare that the research was conducted in the absence of any commercial or financial relationships that could be construed as a potential conflict of interest.

The handling editor declared a past co-authorship with the authors FG and JD.

## Supplementary Material

Supplementary Table 1 Antibody detection by in-house and commercial assays in patients with paired serum and cerebrospinal fluid from Cohort A.
