# Supplementary material for: Limitations of a Commercial Assay as Diagnostic Test of Autoimmune Encephalitis
Source: Front Immunol. 2021 Jun 29;12:691536. doi: 10.3389/fimmu.2021.691536 (PMC8276168; doi:10.3389/fimmu.2021.691536)
Supplement: Supplementary Table 1 — Antibody detection by in-house and commercial assays in patients with paired serum and cerebrospinal fluid from Cohort A. [file Table_1.docx]

| **Patients with paired samples** |  | **Concordant IIFA samples** | **Discordant IIFA**  **samples** |
| --- | --- | --- | --- |
| LGI1+ (15) | serum | 12 | 3 |
|  | CSF | 13 | 2 |
|  |  | **25** | **5*** |
| AMPAR+ (5) | serum | 5 | 0 |
|  | CSF | 4 | 1 |
|  |  | **9** | **1** |
| GABAbR+ (3) | serum | 2 | 1 |
|  | CSF | 2 | 1 |
|  |  | **4** | **2**** |

*from different patients

**from the same patient
